# Arterio-Sclerosis

**Published:** 1907-11-02

**Authors:** John Lindsay Steven

**Affiliations:** Physician and Lecturer on Clinical Medicine, Western Infirmary of Glasgow; Representative of the Faculty of Physicians and Surgeons of Glasgow on the General Medical Council.


### Hospital Clinics.

### ARTERIO-SCLEROSIS.

By JOHN LINDSAY STEVEN, M.D., Physician and Lecturer on Clinical Medicine, Western Infirmary of Glasgow; Representative of the Faculty of Physicians and Surgeons of Glasgow on the General Medical Council.

A Lecture delivered to the Falkirk and District Clinical Association.

Gentlemen,—My first duty is to thank you for the honour you have done me in asking me to deliver to your Association a lecture upon Arterio-Sclerosis. I appreciate the honour very much, but I am somewhat doubtful if, in the course of a lecture, or even many lectures, I can deal in an adequate manner with a subject so intricate in its pathological details, and so far-reaching in the complexity of its clinical manifestations. I can at least, however, attempt to place before you a summary of current opinion upon the etiology and semeiology of this complex disease.

First of all let me impress upon you the great importance of a study of the phenomena of arterial disease, especially in its bearings upon the causation of other organic affections. The health of the bodily organs depends quite as much upon a healthy condition of the arteries as upon a normal state of the circulating fluid itself. Hence, the necessity for clear and accurate views of the nature and cause of arterial diseases if we desire to attain to rational principles in the treatment of these diseases, especially in their early and remedial stages.

One idea which, perhaps more than any other, has dominated the study of arterial disease during recent years is the conception that fundamentally all affections of the arteries are due to the influence of poisons acting deleteriously, though in various ways, on the vessel wall and so producing those changes of structure which we recognise as the clear evidence of vascular disease. Such a conception of the ultimate etiology of arterio-sclerosis commends itself to me very strongly, for I think that recent researches, more particularly those of my colleague Dr. J. M. Cowan, dealing with the pathological histology of arterial disease, go far to prove its truth. Before going further, however, let me recall rapidly one or two elementary points concerning the structure and function of the arteries.

The walls of the arteries are formed of three well-defined coats or layers, which were well known to our great fellow countryman John Hunter*, and his master Cheselden†. The coats of the arteries as now described are the following:—

(*a*) The intima, composed of a layer of endothelium in contact with the blood, a thin subendothelial layer of branched connective tissue corpuscles, and the elastic lamina or fenestrated membrane of Henle.

(*b*) The media, mostly composed of involuntary muscle fibres and yellow elastic tissue, the former predominating in the smaller, the latter in the larger, arteries.

(*c*) The adventitia, composed of connective tissue and elastic tissue, and carrying the vasa vasorum, the vaso motor nerve fibrils, and the lymphatic spaces. A healthy condition of each of these tunics is essential to the health of the artery, and each of them separately or all of them simultaneously may be affected by disease. Integrity of the intima ensures the fluidity of the circulating blood; the elasticity of the larger arteries, such as the aorta, restrains the shock of the ventricular systole and converts the intermittent into a continuous flow, whilst the contractility of the smaller arteries, controlled by the vasomotor nerves supplied to the muscle fibres maintains the tonicity of the vessel and regulates the supply of blood; the adventitia, a sheath of connective tissue, defends the vessel from injury either by trauma or by the extension of contiguous infective processes, and secures the necessary nutrition and innervation of the arterial wall. Bearing these physiological points in mind, many of which were established by John Hunter, we may now proceed to consider the special subject of this lecture—Arterio-Sclerosis.

### Definition and Classification.

To write a definition of arterio-sclerosis is not easy. Etymologically arterio-sclerosis means the thickening and hardening of the walls of an artery. That is all we are entitled to read into the word—that and nothing more. The term is not really the name of a disease, but the designation of a structural lesion resulting from a disease. Logically, therefore, the expression is one which belongs to morbid anatomy rather than to scientific medicine. Yet for good or ill it is now being used as a clinical term, and I fear we must accept it as such and make the best of it. In my earlier days it was used as a synonym of atheroma, and even yet there are many who use the words as if they were interchangeable; but this is no longer possible if we are to have clear ideas of the pathology and clinical bearings of arterial disease. I prefer to take the simple etymological meaning of the term as its definition; to use it in this sense as a general term and to classify varieties. This, I think, is better than to attempt a definition of arterio-sclerosis, which would aim at expressing the whole present signification of the word. Mott, for example, defines arterio-sclerosis as follows: "A local or general thickening of the arterial wall with loss of elasticity, occasioned mainly by fibrous overgrowth of the tunica intima, secondary and proportional to weakening of the muscular and elastic elements of the media."* This is, on the whole, a good definition up to the word "elasticity"; the remainder of it, I think, fails by postulating as essential conditions which are still the subject of controversy. A definition should be a statement of accepted facts, not of opinion.

* "Works." Palmer's edition. Vol. iii., p. 160.

† "Anatomy of the Human Body." Seventh edition, p. 194.

* "Allbutt's System." Vol. vi., 1899, p. 320.

I regard arterio-sclerosis as a term of morbid anatomy, including—1. Focal lesions: (*a*) atheroma or endarteritis deformans; (*b*) endarteritis obliterans. 2. Diffuse or generalised lesions: (*a*) arterio-sclerosis, as used in the present day clinical sense; (*b*) calcareous infiltration of the media; (*c*) arterio-capillary fibrosis of Gull and Sutton.

I purposely exclude such affections as acute arteritis, acute atheroma, septic infection of the arterial wall, etc., as not coming within the scope of my present inquiry. I am prepared to admit that this classification is as faulty as the definition which I have criticised; but to me it presents a working basis for the investigation and treatment of arterial disease and degeneration, which I have found useful.

### Morbid Anatomy and Histology.

It is now necessary that I should speak briefly of the morbid anatomy and histology of arterio-sclerosis. For fuller detail than I can give here on this matter I refer you to the work of Dr. J. M. Cowan,* some of whose excellent lantern slides, taken from his own microscopic sections, I am, by his very kind permission, enabled to use in illustration of this lecture. First of all, let me urge upon you the necessity of keeping in mind that the terms atheroma and arterio-sclerosis do not mean the same thing.

Atheroma affects the larger arteries, such as the aorta, and the smaller arteries, such as those forming the circle of Willis and the coronary arteries of the heart, but very rarely, if ever, involves the medium-sized arteries, such as the radial, the popliteal, etc. It is essentially a patchy lesion, but sometimes the areas are so closely placed as to be practically continuous over a considerable extent of the vessel wall. The change begins in the intima, and is characterised by a hyperplasia of the subendothelial layer of that coat. It thus forms a projection on the internal surface of the artery, and in process of growth presses upon and causes atrophy of the media. Ultimately it undergoes degeneration, fatty or calcareous, and thus gives rise to the atheromatous abscess or calcified patch in the arterial wall. It is frequently the cause of leathery or calcareous dilatation of the aorta, and of aneurysm of that vessel. It may extend to the aortic curtains or may so dilate the orifice of the vessel that the semilunar cusps become incompetent. It may obliterate the orifices of the coronary arteries of the heart, the vessels themselves remaining healthy. Similar changes occur in the cerebral and cardiac coronary arteries, but these being vessels of small size the chief risk is of obliteration from thrombosis.

Endarteritis obliterans is a lesion met with chiefly in arteries of microscopic size, and was first of all described in the arteries of the kidneys in chronic interstitial nephritis. It is also met with as the result of tuberculosis in the arteries of the lung and of the brain. It is characterised by great thickening of the intima, so that the lumen may be endangered and the blood supply to its area diminished, if not entirely cut off. The affected portion of artery may ultimately be converted into a fibrous cord.

* "The Influence of the Acute Infections upon the Arteries." *Glasgow Medical Journal*, August 1906, p. 88.

Such are the focal or patchy lesions of arteries. Let us now consider for a little the diffuse or general affections.

Arterio-sclerosis in its clinical sense is a lesion met with generally in the medium sized arteries, such as the brachial, radial, temporal, femoral, splenic and visceral arteries of the abdomen. As Russell points out it is "due to a thickening of both the tunica media and the tunica intima. The former is due mainly to a hypertrophy of the special cells of that coat, while the latter results from a hyperplasia of the subendothelial tissue."* It involves long lengths of arteries, and is not patchy, as in the case of atheroma or endarteritis obliterans. It is the condition which gives rise to palpable, tortuous and rigid superficial arteries, and it may be present without either atheroma of the aorta, disease in the circle of Willis, or patches in the coronary arteries of the heart. It may also be present without renal disease. Of the coats it is the middle which, in my opinion, is chiefly involved. This coat becomes at first markedly hypertrophied, so that the artery in the earlier stages even has a corded feeling and rolls under the finger.

Calcareous infiltration of the middle coat is a well recognised lesion, quite different from atheroma, and possibly, I think, one of the final results of arterio-sclerosis. It is very likely to be the cause of the extreme hardness and tortuosity met with in the later stages of sclerotic change in the affected arteries whether superficial or splanchnic. Frequently little spicules can be palpated in the walls of such vessels.

Arterio-capillary fibrosis, first described by Gull and Sutton, the third of the varieties of arterio-sclerosis in its etymological sense, according to the scheme I have laid before you, affects chiefly the microscopic arteries of the brain and kidneys, and in this respect at least resembles endarteritis obliterans. It differs, however, in this respect, that the lesion is chiefly a hyperplasia of the external coat. According to Gull and Sutton it is associated with interstitial nephritis, which is, in their view, not a local but a general vascular disease.

### Etiology.

The investigation of the etiology of arterio-sclerosis is much more difficult than that of its macroscopic and microscopic anatomy, because the methods of ascertaining the causation of the condition are much less precise and accurate, dependent as they are upon long continued clinical observations and not upon ocular demonstration. It seems to me that what has to be said upon the causation of arterio-sclerosis may be conveniently ranged under three general headings: (1) Toxic conditions, (2) increased blood pressure, (3) senile change.

(1) Most observers are agreed that toxic agents play a very important part in the production of lesions of the arterial wall; and it is obvious that the poisonous agents may be of the most varied character. The toxins capable of injuriously affecting the arteries may, however, be grouped into two great classes:—

(*a*) *Microbic or biological poisons.*

(*b*) *Organic (metabolic) or chemical poisons.*

*"Encyclopædia Medica." Vol. xiii., p. 348 *et seq.*

(*a*) In the former class we recognise two subdivisions, namely those which may be called specific and those which are non-specific. Among the specific agents we have to deal with the toxins of syphilis, of tubercle, and of the specific fevers. Syphilitic affections of the arteries are chiefly met with in those of the brain and in the aorta; the poison of tubercle is most injurious to the arteries of the lungs and of the brain; and amongst the specific fevers it has been shown that typhoid and scarlet are capable of giving rise to arterial lesions. Non-specific poisons capable of causing arterial disease are generally produced by the various pyogenic organisms. On the whole, I am of opinion that microbic poisons in relation to arterial disease are more likely to produce focal than diffuse lesions, that is to say that atheroma rather than arterio-sclerosis is likely to result from their action.

(*b*) Chief amongst the chemical poisons capable of producing disease of the arteries is to be placed the toxin (or toxins) of uræmia; and the lesion most likely to be produced is a general rather than a focal one. The close relation between renal disease and generalised affections of the arteries has long been recognised, and it is difficult to avoid the conclusion that it is an etiological one. The waste products of an imperfect metabolism must also be considered as potent agents in the production of arterio-sclerosis.

(2) Increased blood-pressure is generally believed to be one of the most powerful causes of arterio-sclerosis, but I am not at all convinced that this is so. Blood-pressure depends chiefly upon two factors, namely the peripheral resistance as regulated by the functional activity of the arterio-capillary wall, and the central propelling force of the cardiac contractions, which are controlled by the functional capacity of the myocardium. Variations of the blood-pressure depend upon the special functional condition of the heart or of the arteries, or of both combined at a given time, and if this be so, then altered blood-pressure must be looked upon rather as a result than a cause of the special changes in the cardio-vascular wall on which the intravascular tension depends. In so far as arterio-sclerosis is concerned, I have endeavoured to show that the exciting cause of the changes of structure in the vessel wall is the presence of toxic agents in the blood which injuriously affect the arterial tissues. If this be true, then it is conceivable that the morbid changes may take place in the arterial wall independently of the blood-pressure, at least as a causal factor. If the blood-pressure depends upon the state of the vessel wall for the time being, and if the condition of the arterial wall both structural and functional is due to the presence of toxic agents, then ultimately the state of the blood-pressure is regulated by the agents acting upon the vessel wall. For these reasons I regard increased blood-pressure not as a cause but rather as a result of these changes in the arterial wall, which ultimately produce the morbid condition which we call arterio-sclerosis.

(3) Senile change, whatever this rather indefinite phrase may connote, I look upon as an important factor in the etiology of arterio-sclerosis. It is, I think, probable that the primary cause of these changes in the tissues which are called senile is again the presence of toxic agents in the blood, and after what has already been said it is unnecessary to dwell further upon the matter.

### Semeiology.

It is with the initial symptoms of the disease that the physician is perhaps chiefly concerned, because it is at this period only that treatment is likely to be most effective. But the initial symptoms are very indefinite because they are dependent upon metabolic changes which are often little known or understood. The early symptoms are most varied, and follow no regular order in their manifestation. Disorders of the gastro-intestinal system are very common at the outset, such as acidity, flatulence, irregularity of the bowels. Along with these signs there is often increasing lassitude, and inability to sustain attention for any lengthened period of time. Frequently muscular pains and cramps are complained of, and some have thought that such pains are really arterial, and produced by the changes going on in the vessel walls. Headaches and sensations of tightness or fullness in the head are common, as well as shortness of breath and uneasy feelings or pain in the epigastrium or behind the lower end of the sternum, the latter being usually experienced after a meal. The uneasy epigastric and sternal sensations are generally associated with flatulence, and are relieved by the expulsion of wind. The pulse is frequently hard, even before manifest pathological changes in the arterial wall have taken place, the increase in tension being due to contraction of the muscular coat brought about by the circulating toxins. Such is an epitome of the rather indefinite and often confusing symptom-groups which mark the onset of arterio-sclerosis, that period when something may be done to arrest the progress of the malady.

The symptoms of the fully developed malady, when the arteries are rigid, hard, tortuous and lime-infiltrated, are usually those of progressive cardiac failure. Dyspnœa and palpitation on exertion, and later orthopnœa and passive congestion are the symptoms which present themselves, and which indicate that the arterial deterioration has at length told upon the structure and function of the cardiac muscle. At this stage treatment can do little but attempt to maintain the flagging energy of the heart.

The effect of arterial disease upon the heart varies according to the variety of the affection of the arteries. In the case of extensive atheroma and calcification of the aorta, especially of the arch, general hypertrophy and dilatation of the heart is produced, often with well marked physical signs of aortic obstruction and regurgitation. Under such circumstances not infrequently the case is looked upon as primarily cardiac, and sometimes it is not until a post-mortem examination has been made that it is realised that the disease has been primarily arterial. In cases of pure arterio-sclerosis—that is, general thickening of the medium sized arteries throughout the body—there is often little or no obvious effect upon the heart, except a very moderate hypertophy of the left ventricle. Sometimes, indeed, the heart may be apparently unchanged, or there may be at the most a slight thinning and flaccidity of the ventricular wall, a condition which may be said to be characteristic of the senile heart. Very different is the effect upon the heart of long-standing renal disease, especially of the interstitial or granular variety. Here hypertrophy confined to the left ventricle without marked dilatation of the cavity is the rule. In such cases there is often no arterio-sclerosis in the sense in which I have used the term in this lecture.

### Treatment.

The treatment of arterio-sclerosis depends entirely upon accurate knowledge, so far as we can gain it, of the pathogenesis of the disease. If, as I have endeavoured to show, the ultimate cause of the disease is metabolic or toxic, then we have a guide to the rational treatment of its earlier manifestations, in which alone we can hope to do any permanent good. The diet and habits of the patient must, first of all, be carefully regulated. Excessive eating must be stopped, and the supply of nitrogenous and highly seasoned foods curtailed. The consumption of alcohol must be strictly moderate, and if circumstances render it necessary, total abstinence should be insisted upon. Tobacco should be used very sparingly, and moderate exercise, short of fatigue, recommended. Mental worry and business anxieties should, as far as possible, be avoided; and in certain cases a prolonged holiday, with a visit to some spa at home or abroad, should be ordered. These, with any other hygienic measures, which may suggest themselves in individual cases, should come first; they are probably of greater importance than drugs. For the earlier gastro-intestinal disturbances grey powder, rhubarb, soda in appropriate doses and at prescribed intervals are serviceable. Mineral aperient waters and salines, such as Carlsbad salts, are also of service. For the reduction of high arterial pressure the nitrites may be employed, and I know of none better than the sweet spirit of nitre. For the actual changes in the arterial wall occasional courses of iodide of potassium, in from 5- to 10-grain doses, are useful.

